# Heterogeneous malaria transmission in long-term Afghan refugee populations: a cross-sectional study in five refugee camps in northern Pakistan

**DOI:** 10.1186/s12936-016-1305-7

**Published:** 2016-04-27

**Authors:** Sobia Wahid, Gillian H. Stresman, Syed Sajid Kamal, Nuno Sepulveda, Immo Kleinschmidt, Teun Bousema, Chris Drakeley

**Affiliations:** Department of Zoology, University of Peshawar, Peshawar, Pakistan; Department of Immunology and Infection, London School of Hygiene & Tropical Medicine, Keppel Street, London, WCIE 7HT UK; Vector Control Department, Merlin Malaria Control Programme, Khyber Pakhtunkhwa, Pakistan; Department of Infectious Disease Epidemiology, London School of Hygiene & Tropical Medicine, Keppel Street, London, WCIE 7HT UK; Department of Medical Microbiology, Radboud University Medical Center, 6500 Nijmegen, The Netherlands

**Keywords:** Malaria transmission, Hotspots of exposure, *Plasmodium falciparum*, *Plasmodium vivax*, Afghan refugees, Pakistan

## Abstract

**Background:**

Afghan refugees in northern Pakistan have been resident for over 30 years and current information on malaria in this population is sparse. Understanding malaria risk and distribution in refugee camps is important for effective management both in camps and on return to Afghanistan.

**Methods:**

Cross-sectional malariometric surveys were conducted in five Afghan refugee camps to determine infection and exposure to both *Plasmodium falciparum* and *Plasmodium vivax*. Factors associated with malaria infection and exposure were analysed using logistic regression, and spatial heterogeneity within camps was investigated with SatScan.

**Results:**

In this low-transmission setting, prevalence of infection in the five camps ranged from 0–0.2 to 0.4–9 % by rapid diagnostic test and 0–1.39 and 5–15 % by polymerase chain reaction for *P. falciparum* and *P. vivax*, respectively. Prevalence of anti-malarial antibodies to *P. falciparum* antigens was 3–11 and 17–45 % for *P. vivax* antigens. Significant foci of *P. vivax* infection and exposure were detected in three of the five camps. Hotspots of *P. falciparum* were also detected in three camps, only one of which also showed evidence of *P. vivax* hotspots.

**Conclusions:**

There is low and spatially heterogeneous malaria transmission in the refugee camps in northern Pakistan. Understanding malaria risk in refugee camps is important so the malaria risk faced by these populations in the camps and upon their return to Afghanistan can be effectively managed.

## Background

Prior to the conflict, Afghanistan had an effective malaria control programme with a focus on vector control, with transmission maintained at very low levels [1, 2]. In 1978, a large number of Afghan refugees migrated to areas in Pakistan, including Khyber Pakhtunkhwa (KP), and settled in camps, some of which are in areas capable of supporting malaria transmission [3]. The influx of a large and immunologically naïve population led to malaria epidemics in the refugee camps in 1997 and 2002 prompting a focus on providing effective malaria control programmes [3–6]. Malaria control programmes established in the refugee camps included distribution of insecticide-treated bed nets (ITNs), indoor residual spraying (IRS) and free malaria testing and treatment [6–8]. The malaria control efforts led to a decline in the reported burden of malaria in the refugee camps and, under control, transmission was maintained at low endemic levels [5, 9].

The state of malaria infection and exposure in this refugee population after residing in northern Pakistan has received little attention in recent times as well as which diagnostic tool is most suited to such low-transmission settings [10, 11]. As health services are scaled down, there is a risk that malaria will re-emerge in the camps [12–14]. Furthermore, as refugees are repatriated to Afghanistan, a country that is rebuilding public health infrastructure, there may be the potential to introduce transmission if individuals are relocated to receptive areas. Conversely, there is a risk of epidemics if a large immunologically naïve population is relocated to an area with ongoing malaria transmission [15].

To provide current data to inform decision-making for managing malaria in this vulnerable population, a malariometic survey was conducted in five Afghan refugee camps in northern Pakistan to: (1) assess the utility of diagnostic tools in this low-transmission setting, including rapid diagnostic test (RDT), polymerase chain reaction (PCR) and the presence of anti-malarial antibodies for *Plasmodium vivax* and *Plasmodium falciparum*; (2) identify factors associated with a current infection as well as exposure to *P. vivax* and *P. falciparum* in a stable refugee population; and, (3) to identify any spatial patterns of malaria present within the Afghan refugee camps in Pakistan.

## Methods

### Study area and sampling

**Fig. 1:**
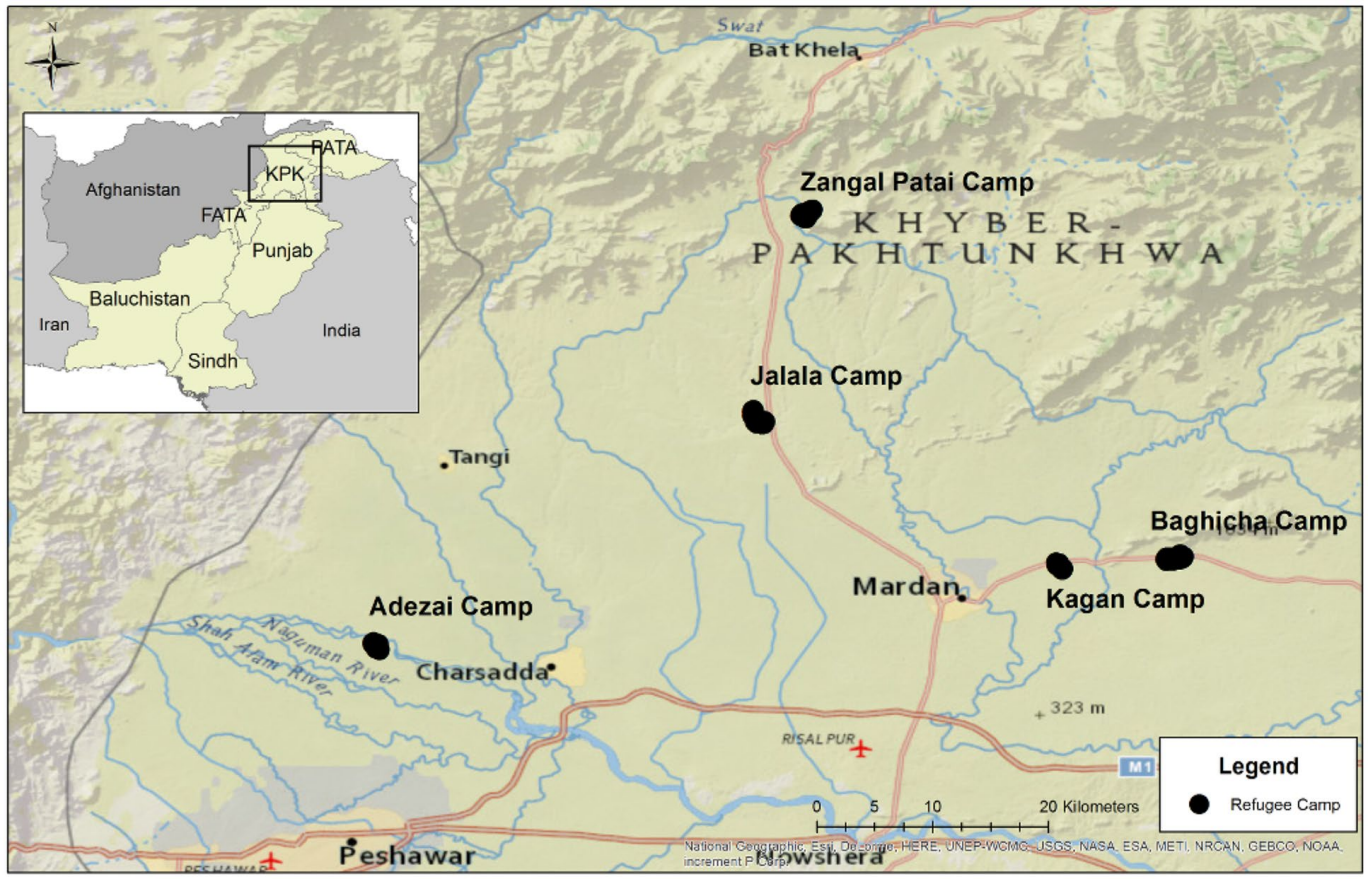
Study area with the location of the five Afghan refugee camps in Pakistan selected for this survey. National Geographic basemap source: Esri, DeLorme, HERE, UNEP-WCMC, USGS, NASA, ESA, METI, NRCAN, GEBCO, NOAA, iPC

Study participants were selected from five Afghan refugee camps in Mardan (Baghecha, Kaghan and Jalala camps) and Peshawar (Adezai) districts in the province of Khyber Pakhunkwa (KPK) in Pakistan, as well as the Zangal Patai camp in the Malakand agency tribal area (Fig. 1). Camps were established in the late 1980s with some refugees being resident for more than 30 years at the time of the survey. The surveys took place between 24 June and 19 September, 2010 to coincide with the main *P. vivax* transmission season and before the *P. falciparum* transmission season, which typically peaks in late October [5, 9, 16]. The main vectors in the area are *Anopheles stephensi* and *Anopheles culicifacies* and the majority of the malaria infections are due to *P. vivax* [4]. The area is characterized by sandy and marshy land and is well irrigated for sugar cane, wheat and rice production. Houses are primarily constructed with rocks, bricks and mud and animal ownership is common. Free primary health care is provided at basic health units (BHUs) established in each camp and run by the UN High Commissioner for Refugees. Malaria testing and treatment of microscopically confirmed cases is provided free according to national guidelines [15].

Sample size calculations for the survey were derived based on estimating anti-malarial antibody seroconversion rates (λ) of 0.01 with a residual standard deviation less than 0.25, which resulted in a sample of three people per household with a minimum of 167 households [17, 18]. A numbered list of all current households was obtained for each camp and the total number of people per age group in each household was recorded to provide a sampling weight for the data. Two-hundred households per camp were randomly selected to allow for refusal and absenteeism. One person from each of three age groups (one to five, six to 20 and >21 years) per household was randomly selected for collection of blood samples.

The household heads in all selected households were approached for written informed consent and questionnaires were administered to collect information on household characteristics, including wealth indices, travel history, malaria control behaviour, and demographic information. Finger-prick blood samples were collected on Whatman 3 mm filter paper (Maidstone, UK) from the selected individuals for subsequent laboratory analysis after written consent was obtained. The CareStart Pf/Pv combo (Access Bio, Inc. NJ, USA) RDT was performed to detect current malaria infections with *P. vivax* (pLDH) and/or *P. falciparum* (HRP2). All individuals found to be RDT positive were referred to a BHU for full evaluation and for appropriate treatment. Blood was also collected onto Whatman 3 mm filter paper (Maidstone, UK) for laboratory analysis.

### Laboratory analysis

Filter-paper blood spots were dried in the field and stored with desiccant at −20 °C and shipped to London for analysis. Antibodies to *P. falciparum* and *P. vivax* Apical Membrane Antigen-1 (AMA-1) and Merozoite Surface Protein-1_19_ (MSP-1) were detected using enzyme-linked immunosorbent assay (ELISA) as previously described [11]. In each camp, individuals positive by RDT and a random selection of 120 RDT-negative individuals were assayed by a nested, species-specific PCR as previously described [19]. Due to the higher malaria prevalence observed in Jalala camp, all samples were analysed by PCR. Briefly, DNA was extracted using chelex-saponin and genus-specific primers were used in the nest-1 reaction and two separate nest-2 reactions were conducted using primers specific for *P. vivax* and *P. falciparum*.

### Statistical analysis

Data analysis was conducted using Stata v12.0 (Stata Corp LP, TX, USA) and R v3.2 (R-Project, USA) statistical software. Duplicate ELISA OD values were averaged and normalized against the positive control sample on each plate. OD data were then converted to antibody titres, expressed in arbitrary units (AU/ml), using a standard curve obtained from hyperendemic control sera. Seropositivity was defined by fitting a mixture model to normalized OD values [20]. The model assumed two Gaussian distributions, one for seronegative values and the other seropositive values. The mean OD plus three standard deviations of the seronegative values for each species and antigen was used as the cut-off value for seropositivity. An individual was considered to be seropositive if they responded to at least one of the two antigens tested for each species [21]. Seroprevalence was stratified into age groups and the seroconversion rate (SCR) was estimated by fitting a reverse catalytic conversion model under a binomial sampling assumption [17]. PCR prevalence was calculated using a bootstrap approach to avoid bias associated with the sampling approach. Briefly, a subset of samples assayed by PCR was randomly selected with replacement according to RDT positivity. PCR prevalence of the sample was determined and repeated 10,000 times. The mean of the bootstrapped estimates provided the overall PCR prevalence per camp and 95 % confidence intervals were calculated according to the Chen-Shao method [22].

Principal component analysis was used to generate a score for socio-economic status (SES) based on household asset ownership data and grouped according to quintiles [23]. Logistic regression was used to assess risk factors for both *P. falciparum* and *P. vivax* using the survey command, weighted for household population size, and adjusting for clustering within camps. Spearman’s rank correlation coefficient was calculated to compare diagnostic tools within camps. Hotspots were determined assuming a Bernoulli model with SatScan software v9.2 (Harvard, Boston, USA). Elliptical and circular windows were used allowing for a maximum spatial cluster size of both 50 and 25 % of the population at risk. Those households showing evidence of a significantly (p < 0.05) increased prevalence compared to the rest of the camp by any of the scans were considered to be part of a hotspot [11, 24]. Separate scans were conducted for sero- and RDT positivity for both *P. vivax* and *P. falciparum* and results were analysed using ArcGis v10.2 (ESRI, CA, USA). Due to the sub-set of samples analysed, spatial analysis was not conducted on PCR results.

### Ethical approval

Ethics approval for the study was granted by both Peshawar University (#02/EC/Pharm) and the London School of Hygiene and Tropical Medicine (#5715). Individual written informed consent was sought from heads of included households, and from all selected participants by signature. Consent for children under the age of 18 was provided by a parent/guardian.

## Results

In total, 2522 people were sampled in 845 households across the five refugee camps (Table 1). Reported bed net use the previous night in camps ranged between 3.2 % (95 % CI 1.6–4.7 %) of those sampled in Jalala camp and 63.7 % (95 % CI 59.5–67.9 %) in Baghicha. Reported IRS in the previous 12 months (≤10.4 %) and fever in the previous 2 weeks (≤10.0 %) was low across all camps.

**Table 1:** Demographics by camp

	Adezai		Baghicha		Jalala		Kagan		Zangal Patai	
	%	95 % CI	%	95 % CI	%	95 % CI	%	95 % CI	%	95 % CI
N										
<5	169/1323		169/1112		167/1205		161/957		169/1597	
5–20	171/1548		170/1961		170/1343		168/1946		168/1590	
>20	167/1632		168/1220		168/1458		167/1232		170/1990	
Sex—% male	39.8	35.6–44.1	40.2	35.9–44.5	35.2	31.1–39.4	41.9	37.6–46.3	47.5	43.2–51.9
Camp resident 6 months	9.1	6.6–11.6	2.6	1.2–3.9	3.2	1.6–4.7	15.1	11.9–18.3	2.2	1.0–3.4
Travel 3 months	14.0	11.0–17.0	14.8	11.7–17.9	11.9	9.0–14.7	11.3	8.5–14.1	21.3	17.7–24.9
Fever 2 weeks	1.8	0.6–3.0	10.0	7.4–12.7	7.1	4.9–9.4	2.8	1.4–4.3	2.6	1.2–3.9
ITN last night	16.0	12.8–19.2	63.7	59.5–67.9	3.2	1.6–4.7	51.4	47.0–55.8	9.3	6.7–11.8
IRS 12 months	4.1	2.4–5.9	2.4	1.0–3.7	7.7	5.4–10.0	2.4	1.1–3.8	10.4	7.8–13.1
SES										
1	22.0	18.4–25.6	21.9	18.3–25.5	29.9	25.9–33.9	21.8	18.1–25.4	16.0	12.8–19.2
2	16.1	12.8–19.3	18.3	15.0–21.7	8.9	6.4–11.4	27.0	23.1–30.9	12.4	9.5–15.3
3	19.0	15.6–22.5	30.4	26.3–34.4	11.3	8.5–14.0	16.9	13.6–20.2	11.8	9.0–14.6
4	20.8	17.3–24.4	18.9	15.5–22.3	23.2	19.5–26.8	22.4	18.7–26.0	19.5	16.1–23.0
5	22.0	18.4–25.6	10.4	7.8–13.1	26.7	22.8–30.6	11.9	9.0–14.7	40.2	35.9–44.5

*N* The number of people sampled/the total number of people in sampled households

Estimates of malaria infection and exposure were consistent with low endemicity in all camps (Table 2). Seroprevalence for *P. vivax* ranged from 47.5 % (95 % CI 43.1–51.9 %) in Jalala camp and 17.6 % (95 % CI 14.2–20.9 %) in Adezai. Similarly, PCR prevalence was highest in Jalala with 15.6 % (95 % CI 12.5–18.6 %) for *P. vivax* malaria and the lowest in Kagan with 3.7 % (95 % CI 0–6.2 %). *Plasmodium vivax* infection by RDT was lower than PCR: Jalala camp reported the highest RDT prevalence at 9.7 % (95 % CI 7.1–12.3 %) and lowest was in Adezai camp (0.4; 95 % CI 0–0.9 %). Overall, *P. falciparum* infection and exposure was lower than that of *P. vivax*. Seroprevalence estimates ranged from 9.9 % (95 % CI 7.2–12.5 %) in Zangla Patai to 2.4 % (95 % CI 1.1–3.8 %) in Kagan refugee camp. Evidence of *P. falciparum* infection by PCR was only observed in Jalala (1.4, 95 % CI 0.6–2.6 %) and Kagan (0.8, 95 % CI 0–2.3 %) camps and by RDT in Zangal Patai (0.2, 95 % CI 0–0.65 %).

**Table 2:** Malaria outcomes per camp

	Adezai		Baghicha		Jalala		Kagan		Zangal Patai	
	%	95 % CI	%	95 % CI	%	95 % CI	%	95 % CI	%	95 % CI
*P. vivax*										
SCR	0.016	0.013–0.020	0.036	0.031–0.043	0.063	0.054–0.073	0.029	0.025–0.035	0.017	0.014–0.021
Seroprevalence	17.6	14.2–20.9	32.5	28.4–36.6	47.5	43.1–51.9	28.9	24.9–32.9	19.1	15.7–22.6
PCR	7.0	3.7–11.7	9.1	4.4–13.9	15.6	12.5–18.6	3.7	0–6.2	6.9	2.8–10.6
RDT	0.4	0–0.9	2.8	1.3–4.2	9.7	7.1–12.3	2.0	0.8–3.2	4.1	2.4–5.9
*P. falciparum*										
SCR	0.002	0.002–0.004	0.007	0.005–0.009	0.003	0.002–0.005	0.002	0.001–0.003	0.008	0.006–0.011
Seroprevalence	3.2	1.6–4.7	9.1	6.6–11.6	4.5	2.7–6.4	2.4	1.1–3.8	9.9	7.2–12.5
PCR	0		0		1.4	0.6–2.6	0.8	0–2.3	0	
RDT	0		0		0		0		0.2	0–0.6

*Plasmodium vivax* transmission intensity, as estimated by SCR, followed a similar pattern to other infection metrics in that it was comparatively low in all camps with the highest SCR observed in Jalala camp (0.062, 95 % CI 0.054–0.073) and lowest in Adezai (0.016, 95 % CI 0.013–0.020) (Table 2). Even with the small sample size, there was a moderate correlation between the ranking of camps transmission intensity according to *P. vivax* SCR and both PCR (r = 0.6; p = 0.28) and RDT (r = 0.7; p = 0.19). PCR bootstrapping estimates correctly classified Jalala as having the highest transmission but Adezai was estimated to have higher PCR prevalence than both Kagan and Zangal Patai despite the lowest SCR. SCR estimates for *P. falciparum* suggest residual very low level exposure is occurring in all camps, however despite an indication of limited transmission, Jalala and Kagan as well as Zangal Patai had evidence of current infection based on PCR and RDT.

In adjusted analysis for factors associated with *P. vivax* RDT positivity, adults over 20 years of age were significantly less likely (OR 0.29, 95 % CI 0.15–0.57) to have an infection compared to children under 5 years of age (Table 3). Reduced prevalence of being infected with *P. vivax* was seen in those who had been living in the camp for the last 6 months compared to arriving in the camp more recently (OR 0.18, 95 % CI 0.05–0.71). Those reporting a fever in the previous 2 weeks had over seven times the odds (OR 7.03, 95 % CI 3.47–14.26) of being infected compared to those not reporting a fever. Both reported use of an ITN the previous night and use of mosquito repellants in the household were associated with a reduced odds of having a *P. vivax* infection while those of higher SES were also less likely to be infected (Table 3).

**Table 3:** Factors associated with *Plasmodium vivax* infection by RDT

	RDT			
	Univariate		Multivariate	
	OR	95 % CI	OR	95 % CI
Age group (years)				
≤5	1	–	1	–
6–20	0.80	0.50–1.28	0.71	0.42–1.20
>20	0.36**	0.20–0.68	0.29*	0.15–0.57
Sex—male	1.64	0.99–2.69		
Camp resident 6 months	0.14***	0.03–0.67	0.18***	0.05–0.71
Travel 3 months	1.02	0.53–1.94		
Fever—2 weeks	6.35*	3.57–11.31	7.03*	3.47–14.26
Sought malaria treatment	2.48***	1.13–5.47		
IRS 12 months	0.41	0.10–1.59		
ITN last night	0.18**	0.07–0.47	0.14*	0.05–0.39
Use repellants	0.30*	0.17–0.50	0.29*	0.17–0.50
Roof—iron	0.36**	0.19–0.68		
Eaves—closed	0.54***	0.30–0.98		
Animal ownership	1.28	0.77–2.14		
SES				
1	1	–	1	–
2	0.39***	0.16–0.95	0.43	0.18–1.06
3	0.59	0.28–1.25	0.62	0.26–1.46
4	0.48***	0.24–0.97	0.55	0.28–1.10
5	0.15*	0.07–0.32	0.15*	0.07–0.32

* p < 0.0001, ** p < 0.01, *** p < 0.05

As expected, both *P. vivax* and *P. falciparum* seroprevalence increased with age: those 20 years of age and older had 7.28 (95 % CI 5.61–9.45) and 2.73 (95 % CI 1.69–4.39) the odds of being positive for anti-malarial antibodies to *P. vivax* and *P. falciparum*, respectively, compared to those younger than 5 years. Those reporting fever in the previous 2 weeks also had increased odds of being seropositive for both *P. vivax* (OR 3.29, 95 % CI 2.20–4.91) and *P. falciparum* (OR 1.99, 95 % CI 1.05–3.77). Reported use of mosquito repellents was associated with a 30 % reduction in odds of exposure for both species (Table 4). Being a resident in the camp for the 6 months prior to the survey was associated with a significant reduction (OR 0.15, 95 % CI 0.05–0.42) whereas having reported to have travelled in the past three months was associated with increased *P. falciparum* seroprevalance (OR 1.99, 95 % CI 1.05–3.77). Living in a household with an iron roof was associated with an increase in odds with being seropositive to *P. falciparum* (OR 1.63, 95 % CI 1.04–2.55). Owning animals showed a 1.63 (95 % CI 1.30–2.05) increase in the odds of being seropositive for *P. vivax* compared to not owning any animals but had no association with *P. falciparum*.

**Table 4:** Factors associated with *Plasmodium vivax* and *Plasmodium falciparum* seropositivity as a marker for exposure

	*P. vivax*				*P. falciparum*			
	Univariate		Multivariate		Univariate		Multivariate	
	OR	95 % CI	OR	95 % CI	OR	95 % CI	OR	95 % CI
Age group (years)								
≤5	1	–	1	–	1	–	1	–
6–20	1.89*	1.44–2.47	1.91*	1.44–2.53	1.93***	1.15–3.23	1.87***	1.11–3.15
>20	7.28*	5.61–9.45	7.67*	5.83–10.10	3.05*	1.90–4.91	2.73*	1.69–4.39
Sex—male	0.60*	0.49–0.74			1.02	0.70–1.48		
Camp resident 6 months	0.29	0.08–1.13			0.11*	0.04–0.28	0.15*	0.05–0.42
Travel 3 months	1.93*	1.50–2.49			1.95**	1.31–2.91	1.55***	1.04–2.29
Fever 2 weeks	3.18*	2.21–4.57	3.29*	2.20–4.91	2.03***	1.08–3.82	1.99***	1.05–3.77
IRS 12 months	1.30	0.78–2.14			1.51	0.60–3.79		
ITN last night	0.88	0.23–1.08			1.36	0.87–2.11		
Use mosquito repellants	0.69*	0.58–0.81	0.68*	0.56–0.82	0.64***	0.44–0.91	0.65***	0.45–0.94
Sought malaria treatment	3.47*	2.33–5.17			2.06***	0.99–4.24		
Roof—iron	0.91	0.73–1.15			1.58***	1.02–2.45	1.63***	1.04–2.55
Eaves—closed	0.92	0.74–1.14			0.70	0.45–1.06		
Animal ownership	1.58*	1.29–1.93	1.63*	1.30–2.05	0.86	0.57–1.30		
SES								
1	1	–			1	–		
2	0.77	0.56–1.06			0.90	0.47–1.71		
3	1.03	0.76–1.41			1.03	0.55–1.92		
4	1.20	0.89–1.61			0.68	0.34–1.36		
5	0.96	0.71–1.31			1.35	0.73–2.50		

* p < 0.0001, ** p < 0.01, *** p < 0.05

**Fig. 2:**
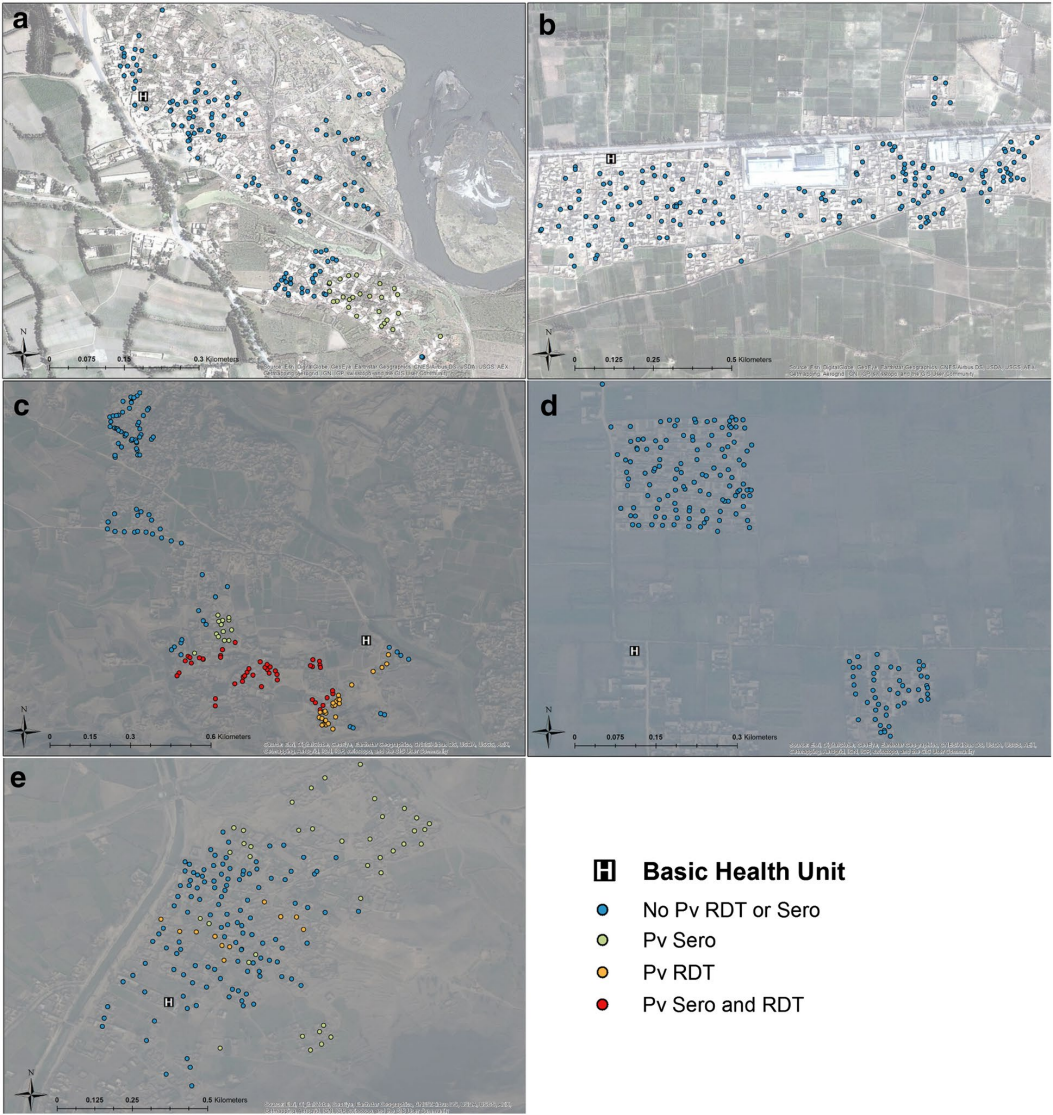
Results of spatial clustering using multiple outcomes. For *Plasmodium vivax* (seropositivity, RDT positivity) for Adezai (**a**), Baghicha (**b**), Jalala (**c**), Kagan (**d**), and Zangal Patai (**e**) refugee camps as estimated from SatScan. *Blue dots* are households that were not part of a hotspot; *green* and *orange dots* were part of a significant hotspot of seropositivity and RDT, respectively; *red dots* were households found to be part of a hotspot using both sero- and RDT-positivity. World Imagery basemap source: Esri, DigitalGlobe, GeoEye, i-cubed, USDA, FSD, USGS, AEX, Getmapping, Aerogrid, IGN, IGP, swisstopo, and the GIS User Community

**Fig. 3:**
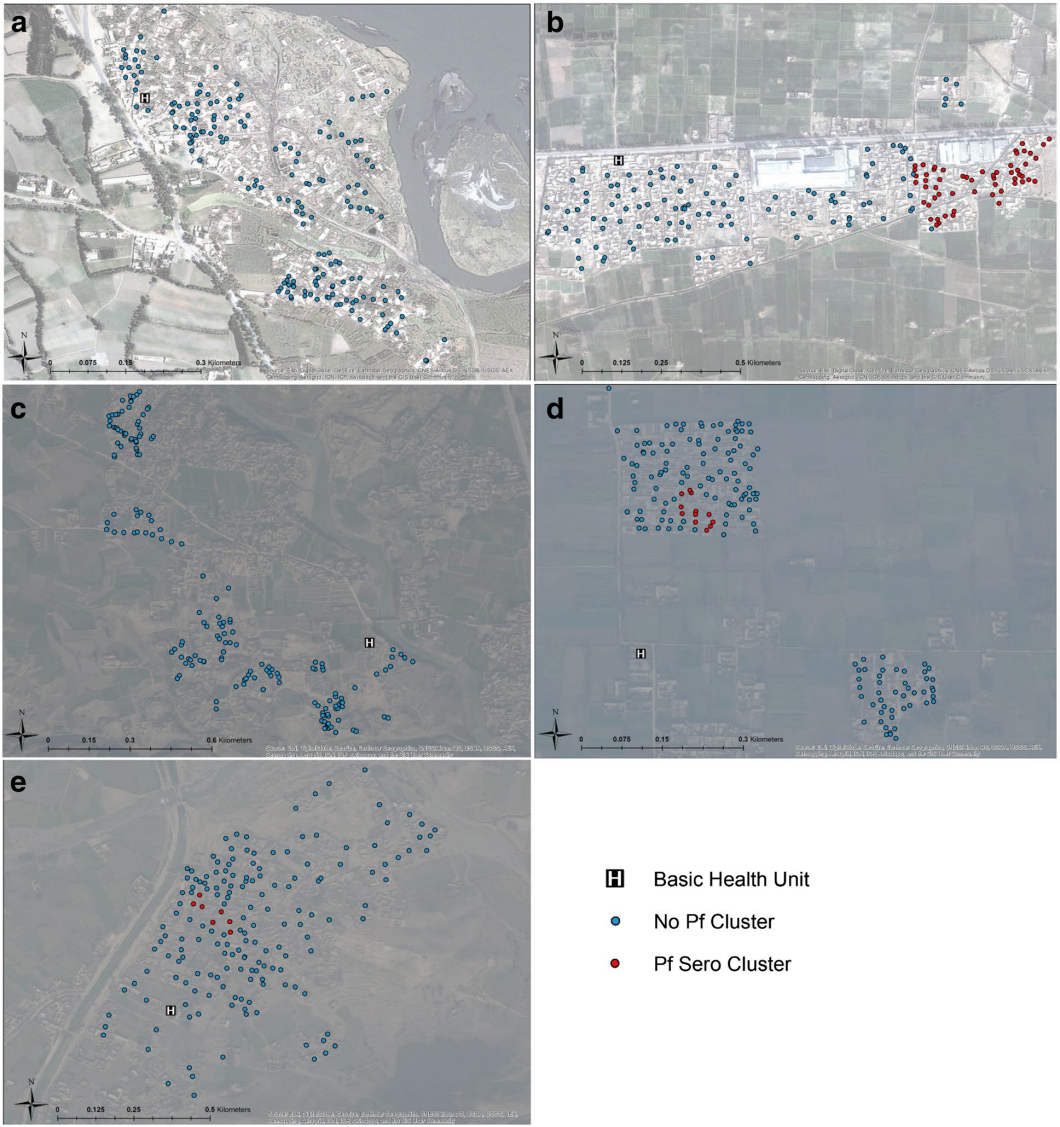
Results of spatial clustering using multiple outcomes. For *P. falciparum* (seropositivity) for Adezai (**a**), Baghicha (**b**), Jalala (**c**), Kagan (**d**), and Zangal Patai (**e**) refugee camps as estimated from SatScan. *Blue dots* are households that were not part of a hotspot and *red dots* were households found to be part of a hotspot of seropositivity. World Imagery basemap source: Esri, DigitalGlobe, GeoEye, i-cubed, USDA, FSD, USGS, AEX, Getmapping, Aerogrid, IGN, IGP, swisstopo, and the GIS User Community

Evidence of spatial clustering of both infection and exposure to *P. vivax* was observed in three of the five refugee camps (Fig. 2). In Jalala camp, where estimates of *P. vivax* transmission was the highest, 51.3 % of sampled households were found to be part of a hotspot by at least one metric and 17.8 % of households showing evidence of significant clustering for all markers (Fig. 2c). Baghicha (Fig. 2b) and Kagan (Fig. 2d) showed no evidence of spatial clustering for *P. vivax* whereas hotspots consisting of 14.8 % of households in Adezai (Fig. 2a) and 28.4 % in Zangal Patai (Fig. 2e) were identified. Evidence of clustering of *P. falciparum* seroprevalence (Fig. 3) were observed in Bachicha (Fig. 3b), Kagan (Fig. 3d) and Zangal Patai (Fig. 3e) camps with hotspots comprising of 34.5, 7.1 and 4.1 % of sampled households, respectively.

## Discussion

This study shows evidence of comparatively low and highly heterogeneous transmission for both *P. vivax* and *P. falciparum* in five Afghan refugee camps in Pakistan. *Plasmodium vivax* infections were detected in all camps by both RDT and PCR and ranked camps in order of transmission intensity similarly to the order quantified by SCR. No *P. falciparum* infections by either RDT or PCR were detected in two of the camps although there was evidence of low transmission detected through serological tools. The limited sensitivity of diagnostic tools for current infection observed in this very low-transmission setting is consistent with other studies and further highlights the importance in having combined diagnostic approaches to ensure that current infections as well as transmission potential are included in decision-making [11, 25]. In the camps in this study, combining sensitive PCR with serological methods generated a more complete picture of where transmission is occurring and thus areas of risk that control programmes might effectively target [10].

Several factors associated with infection and exposure to *P. vivax* as well as exposure to *P. falciparum* were identified in this stable refugee population. The use of mosquito repellants, such as sprays or coils, were consistently associated with a reduction in odds of both infection and exposure. This finding may be related to the limited reported use of IRS and ITNs in some of the camps and conversely, the availability and ease of use of repellents. It may also reflect increased awareness of malaria by those that use repellents. Continuing to promote and ensure the availability of mosquito repellents in addition to ITNs or providing these tools as part of a repatriation package upon returning to Afghanistan could be a useful and simple addition to malaria control programmes [26].

Residing in the camp for the previous 6 months was associated with a significant reduction in the odds of malaria exposure whereas recent travel showed increased odds. These findings suggest that importation of malaria from other areas may be contributing to the maintenance of malaria transmission within these camps [27]. This study did not collect the location of travel, which would be useful in ascertaining whether travel is to high-risk areas or if this association is confounded by other factors. The role of importation sustaining transmission in low-endemic areas has been observed in other areas. For example, in Swaziland, a recent survey found that a single *P. falciparum*-infected individual had reported recent travel to Mozambique where malaria transmission is still high [28]. Additional risk factors such as age, regions or villages visited, or employment type, associated with importation as well as the potential for initiating transmission chains would help to guide effective control programmes in these populations [29]. Furthermore, how to most effectively target this population as well as those at high risk for sustaining transmission (e.g. household members of travelers) as well as the impact of such efforts could provide useful strategies and should be explored. Similarly, by analysing travel behaviour and identifying time and location of most frequent travel could provide novel population for which to target interventions.

Owning animals was associated with increased odds of being seropositive for *P. vivax* but was not associated with exposure to *P. falciparum*. Different *Anopheline* vector species have been identified in Afghan refugee camps and it is likely that they have different blood-feeding behaviours [8]. However, the lack of association to *P. falciparum* exposure may also be due to low levels of seroreactivity observed which is likely a product of the low levels of infections, but also due the differences in the timing of sampling in relation to the sampling with the survey taking place after the main *P. vivax* season and before the *P. falciparum* one.

Finally, a history of fever was associated with both increased odds of *P. vivax* infection and exposure to *P. vivax* and *P. falciparum*. The association with malaria is well known and serves as a useful symptom with which to identify malaria infection. Targeting efforts to areas with a high occurrence of fever could provide a simple indicator to initiate re-active case detection, or targeting populations reporting a higher incidence of fever. However, due to the non-specific nature of using fever to diagnose malaria, particularly in low transmission settings, this approach would need to be validated or be accompanied by a malaria diagnostic tool to confirm infection.

There are a number of limitations to this study. Firstly, the camps were sampled at different time points in the transmission season with Baghicha and Kagan sampled before and Adezai, Jalala and Zangal patai sampled during the main *P. vivax* transmission season. This may have had some impact on the ability to correctly rank transmission intensity with the different metrics. RDT positivity for example, may persist after infection and this will be more likely to occur at the end of the transmission season. However, the low seroprevalence and SCR suggest that seasonal effects on prevalence will be minimal.

The very low *P. falciparum* prevalence may have been the result of sampling all camps prior to the main transmission season, which typically occurs in October [5]. However, the ability to correctly distinguish areas with transmission potential during the period of low transmission is likely more powerful to detect residual foci that would seed the upcoming transmission season and therefore provides valuable insight.

PCR could only be conducted on a sub-set of samples due to time and financial constraints. Whilst bootstrapping techniques were used to obtain population level prevalence estimates, these could not be used to assess risk factors or spatial patterns of infection. The factors associated with current infection are not expected to have varied significantly as findings are consistent with previous studies in the Afghan refugee population as well as other studies in low transmission settings [8, 30, 31]. For example, a study by Sangoro et al. showed that in an epidemic setting, people not using repellents had eight times the odds of being infected, and ITN use has long been associated with protection from malaria [32, 33]. Finally, in the case of *P. vivax* it is unclear if the infection and exposure dynamics described are the result of *de novo* infection or transmission maintained by hypnozoite-derived infections. This is common with most studies on *P. vivax* as there is no diagnostic to identify hypnozoite infections. It is possible that elevated antibody levels in the absence of an infection could be used to indicate the possibility of hypnozoite carriage but this needs to be validated in individuals with known relapses, and with probability a broader range of antigenic targets.

This study suggests that there are low levels of both *P. vivax* and *P. falciparum* transmission occurring in the refugee camps included in this survey. However, although risk is low, it is still higher than reports from the rest of KPK, which suggest 1 % prevalence by RDT [34, 35]. The higher levels of malaria risk observed in the camps is likely a factor of where the camps were located, in areas known to support malaria transmission [3]. Provincial estimates, including a larger range of regions of both rural and urban areas, would mask heterogeneity of transmission and bias estimates towards the null. Recent reports of emerging multidrug-resistant *P. falciparum* strains in Pakistan [36] as well as reports of the presence of sub-standard anti-malarial drugs in Afghanistan are cause for concern [37]. The proportionally large amount of infected individuals in the refugee camps and the proposed repatriation of all refugees to Afghanistan by the end of 2015 could result in a large movement of parasites to a setting that may exacerbate the problem and fuel the spread of multidrug-resistant malaria in the region [38].

## Conclusions

Ultimately, understanding the epidemiology behind malaria transmission in this vulnerable population is essential so that malaria control programmes can effectively target their resources where they can be most effective [39]. As health services in the refugee camps are scaled back and the refugee populations continue to move back to their native Afghanistan, understanding the risk to this migrant population of malaria infection and the spread of parasites conferring markers of drug resistance is paramount so that a fragile public health infrastructure will not be overwhelmed.
